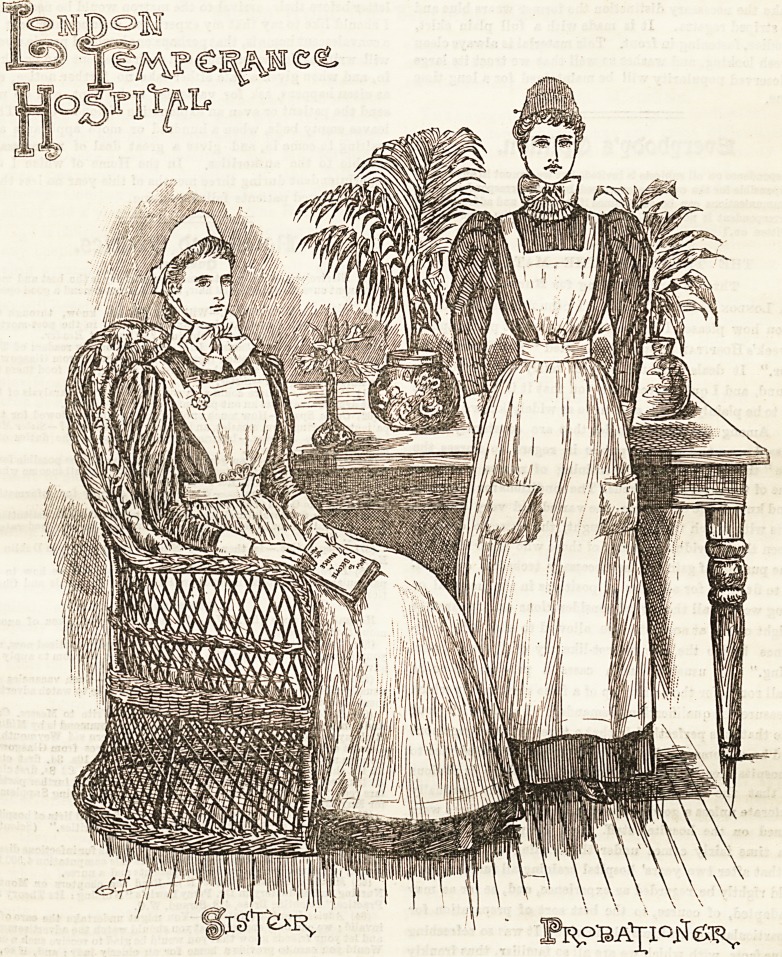# "The Hospital" Nursing Mirror

**Published:** 1896-06-06

**Authors:** 


### "The Hospital" Nursing Mirror.

Being the Extra Nursing Supplement of "The Hospital" Newspaper.

[Contributions for this Supplement should be addressed to the Editor, The Hospital, 428, Strand, London, W.C., and should have the word "Nursing" plainly written in left-hand top corner of the envelope.]

### News from the Nursing World.

### NURSES IN AMERICA AND PRINCESS MAUD'S PRESENT.

We are sorry to hear that some English nurses in America, who belong to the Royal National Pension Fund, were disappointed that special mention was not made of their contributions towards Princess Maud's wedding present. English Pension Fund Nurses who are scattered all over the world joined in the gift, and it was not thought necessary in every case to particularise localities; but it cannot fail to please the Princess to know that those who are working far away from their English home participated not only in the purchase of the present, but in the hearty good wishes that accompanied it.

### NEWS FROM GUY'S.

The Governors of Guy's Hospital have awarded a yearly pension of £36 to Miss Gore, late Sister Patience, on her retirement from the hospital, where she has worked for the last twenty-five years. Her place in Patience Ward has been taken by Miss Stowell. Miss M. Rose (Sister Lydia) has also left the hospital for other work, and is succeeded by Miss Mabel Hobhouse. Societies and clubs are energetically organised at Guy's for the nursing staff. There is a Nurses' Choral Society, which has had a very successful season, the last meeting being held a week or two ago. Now tennis is in the ascendant, and the members of the Nurses' Tennis Club have begun practice on the courts at Honor Oak Park, which, by permission of the Council of the "Clubs' Union," the nurses are at liberty to use three mornings a week.

### "MATRONS' CHRONIC."

"In making a study of hospitals and visiting them," said Mr. Sydney Holland, in his recent paper on a central hospital board, "I have been amazed to find the utter want of knowledge at hospital A of what is done at hospital B, to find that, though year after year extraordinary differences are pointed out in the management of hospitals on matters of the first importance, yet that each one goes on happily in its own course. . . . The secretaries do know a great deal of what goes on at other hospitals, but the matrons as a rule do not, and those of them who have not got the disease I call 'matrons' chronic' (a disease which is complete satisfaction with their own surroundings) seem to suffer from despair and hopelessness of getting their committees to carry out necessary reforms." Mr. Holland's surprise at this curious spirit of self-satisfaction, or indifference, will be shared by many people who know something of the inner life of hospitals. The remark may often be heard, "I have enough to do to manage my own nurses. I have no time or wish to inquire into the customs of other hospitals." It is a pity that this way of looking at the matter should prevail, for if there are many details in which each matron must gain her own experience unaided, there are many points upon which a knowledge of other people's views might be exceedingly helpful, and an ostrich-like attitude can only be harmful and antagonistic to true progress. "Matrons' chronic" is a disease which is unworthy of educated and enlightened women, but it is, unfortunately, impossible to deny that in some measure it does exist.

### TWO TRAGEDIES.

Two wholesale disasters have made the past week memorable for an appalling sacrifice of human life. The populous city of St. Louis, in the State of Missouri, U.S.A., has been devastated by a cyclone, killing over a thousand people, and the coronation of the Tzar has been verily celebrated in blood at Moscow by the calamity of Saturday, when nearly two thousand people were crushed to death on the Chodinsky Plain, in the struggle and fight to reach the booths from whence presents were to be distributed. The terrible part of the Russian catastrophe is that with the most ordinary precautions it would never have happened, but absolutely none appear to have been taken to prevent crowding, which could not have failed to end in a panic. Sixty-nine of the injured victims have been taken to the Marie Hospital where they were visited by the Tzar and Tzaritsa on Monday.

### A DIFFICULTY SOLVED.

Mr. William Rathbone has come to the relief of the deadlock at the Ladies' Charity and Lying-in Hospital, Liverpool, with a proposal which has been accepted by the board of management and the ladies' committee, "in order to effect an amicable settlement, and out of respect to the suggestions emanating from so impartial and competent an adviser." The terms submitted by Mr. Rathbone both to the president of the Medical Institution and the hospital authorities, for the future working of the hospital, are: "That the medical officer on duty at the hospital should have sole and entire medical charge of all patients in the hospital, but it is understood that the matron-midwife, as before, shall have charge of the cases of natural labour, summoning, when requisite, the medical officer on duty, who shall have the right to exercise general supervision over all the cases, and take charge of any patient in whom he detects anything abnormal." The new medical staff will therefore be elected upon these lines.

### ROYAL SOUTH HANTS INFIRMARY.

The medical staff of the Royal South Hants Infirmary have recently passed a resolution strongly condemning the present "insanitary condition" of the hospital, to which they attribute the past "numerous outbreaks of preventible disease . . . among the patients, and more especially among the resident staff, nurses, and servants of the institution." A scheme for the reconstruction of the hospital is now in hand, and, in view of this very serious condition, will doubtless be pushed forward and carried out promptly and thoroughly. The estimated cost is £19,000, and of this sum £5,800 is already promised, though an appeal to the public has only just been issued.

### PRIVATE NURSES AND THE PUBLIC.

Many complaints are made, not always, it must be confessed, without reason, of the ways of private nurses by those who have had to fall back upon their ministrations in times of illness. "Anything rather than a trained nurse in the house, to upset the servants and make work," is the remark constantly heard when the need for skilled help is suggested, and there are too many members of the profession who have brought this reputation upon themselves by a sad want of tact and adaptability. But there is another side to the shield. The private nurse constantly finds herself among people whose demands upon her physical and mental powers are absolutely unconscionable; who expect her to be on duty night and day, for thirty-six hours at a stretch, in an emergency, with only broken rest to follow; to be always bright and cheerful, and to need not a single hour to herself in all the day; to do, in fact, more than mortal woman can do without severely suffering for it afterwards. We speak at the moment from personal knowledge of a case where service of this description has been expected from a nurse for a period of several months. The inhumanity of such treatment needs no comment. But the result is terribly serious for the nurse, who cannot afford to quarrel with her bread and butter, and who yet knows well that a breakdown will probably follow, with subsequent loss of time and money. It is little wonder that after a few experiences of this kind nurses are on the defensive in their relations with the public.

### PROGRESS AT PAISLEY.

The Parish Council of Paisley have carried out a necessary reform in the provision for the sick poor of the district. Fifty beds have been added to the Parochial Hospital, and the patients from the Burgh Poorhouse transferred to that institution, where they will now be cared for by trained nurses instead of by unskilled attendants.

### NURSES' INSTITUTE, BEDFORD.

Bedford seems to do exceedingly little for its poor in the matter of district nurses. According to the annual report of the Bedfordshire Hospital Trained Nurses' Institute a second district nurse is badly wanted, but for lack of an additional £80 a year the idea has had to be given up. Mr. Whitbread, at the annual meeting, remarked that "he should feel a little ashamed of their efforts if they were not able to do that in a town of the size of Bedford," and went on to suggest that to "vitalise their efforts" more opening out in the direction of district nursing was needed "by getting into communication with the ministers of churches of all denominations." Ultimately a small committee was appointed to consider what was to be done in the matter, and we hope their efforts will be well responded to, and the establishment of a second nurse quickly accomplished. The institute in other respects shows a satisfactory record, and the private nurses have had a busy year. The council, in their report, tendered especial thanks to Mrs. Rawson, the lady superintendent, to whose "untiring energy" the institute owes much.

### DISTRICT NURSING IN BIRMINGHAM.

A district Nurses' Home has been started in the parish of St. Thomas', Birmingham, a new development which, it is hoped and expected, will prove of the greatest value. The Home will be at 79, Ryland Road, and Mrs. Butcher, for many years sister at Charing Cross, Walsall, and Cheltenham, has undertaken the work of superintendence. Miss H. E. Bothamley, "Sister Hope," has been appointed head nurse.

### NURSES AND HOSPITAL SATURDAY IN LIVERPOOL.

A letter appeared in the *Liverpool Courier* the other day urging that the proceeds of the forthcoming street collections in aid of the Hospital Saturday Fund in Liverpool might be augmented by the nurses' "presence in attendance at the collecting boxes, wearing the picturesque garb of their humane calling!" Street collections are at all times to be deplored as a mode of raising money, and nothing could be more undesirable than for nurses to be thus publicly associated with street begging. We certainly hope that the authorities of the Liverpool hospitals did not allow the members of their nursing staffs to masquerade the town in a uniform which is unfortunately already in considerable disrepute owing to the questionable people who, as matters stand, can and do with impunity, don a dress to which they have no right.

### MEDICAL WOMEN AT THE MELBOURNE HOSPITAL.

The Melbourne Hospital Committee have lately elected two ladies as resident medical officers. The resident staff for the year is usually chosen from the first six names on the list at the final honour examinations of the University, the committee retaining the right of passing over any applicant considered unsuitable. In this year's list amongst the first six were the names of Miss Ælfreda Hilda Gamble and Miss Janet Stocks Greig, both ladies claiming their right of election. There was some discussion, in the course of which doubts were expressed by one or two members of the committee as to the success of the experiment, chiefly on the ground that the matron and the nurses as well as the medical staff did not like the change; finally a division was taken, and Miss Gamble and Miss Greig were elected by 13 votes to 5. This is the first time in the history of Victoria that women have been appointed to the post of resident medical officer.

### SHORT ITEMS.

The next examination of the Society of Trained Masseuses will be held on Wednesday, July 1st, at six o'clock, at the Trained Nurses' Club, 12, Buckingham Street, Strand.—At the Sarah Acland Nursing Home, Oxford, a room has been recently endowed for the benefit of women students and ladies of small means. Board, lodging, and nursing free of charge are offered to the occupants of the Rose Room. Applicants should apply to "Rose," care of Lady Superintendent. —A sacred concert was held in Darlington Public Park on the Queen's birthday in aid of the Darlington branch of the Q.V.J.I.N.—The Red Cross Sisterhood, which owed its foundation in the first instance to the late Empress Augusta, the Empress Frederick, and the late Grand Duchess Alice of Hesse at the time of the Franco-German war, has just been celebrating its twenty-fifth year of active nursing work in time of war.

### Hygiene: For Nurses.

By JOHN GLAISTER, M.D., F.F.P.S.G., D.P.H.Camb., Professor of Forensic Medicine and Public Health, St. Mungo's College, Glasgow, &c.

### IX.—HEAT IN RELATION TO HEALTH—(*Continued*).

COMPARATIVE VALUES OF HEAT-GIVING SOURCES.—Conduction is the mode by which heat is propagated in solid bodies. If one end of a long knitting needle be held in a flame, sooner or later the heat will travel along the wire to the fingers. Here the heat is transmitted from one metallic particle to the other, until the whole length is traversed. Bodies differ as to their capacity of conducting heat. This is called *conductivity*. Some are good, some bad, conductors. A good conductor is one which robs a body warmer than itself quickly of its heat; a bad conductor, slowly; the former parts with its heat readily, the latter, grudgingly. The sensations, as has been said, are but poor guides to temperature. For example, if the hands be put, in succession, on the fire-irons, the stone hearth, the wooden floor, and a a carpet, we are apt to conclude that they vary in coolness in the order named. But a thermometer registers the same temperature for each. Whence, then, the fallacy of the senses? The conductivity of the substances named vary in the order named, the first being the best, and the last the worst, heat conductor. The sensation of cold when the hand is on the iron, is due to the rapid absorption of the heat of the hand by the metal. And what the hand loses, the metal receives; and a thermometer will now register a higher temperature in the iron than before the experiment. It is for this reason that door handles are made of wood, or porcelain, in preference to metal, and that hot-water pipes or engine fittings are sometimes covered by felt, or the teapot is wrapped in a "cosy," or, in our ice-making apparatus, the outer vessel is of wood, and the inner of metal.

SOURCES OF HEAT IN HOUSES.—These are (1) open coal or wood fires; (2) closed stoves (coal or oil); (3) gas fires; (4) hot-water pipes (at ordinary pressure); (5) hot-water pipes (at high pressure); and (6) steam pipes. The open coal fire still holds, as it is likely to maintain, the premier position as the source of heat in our rooms. It sends out radiant heat which is stimulating and exhilarating. To some extent, doubtless, sentiment prejudices mankind in favour of the open fire. But, apart from this, there is much to be said in its favour. It least of all directly warms the room-atmosphere; its radiant heat warms the objects which it strikes—the bodies of the occupants, the furniture, the walls—and the whole room is thereby warmed. The chief objection to our present mode of utilising the heat is needless waste. In an ordinary fireplace, from three-fourths to five-eighths of the total heat generated is of no value to the apartment. It is usually considered lost, but this is not so, as it heats the walls of the house—thereby keeping them dry—and it raises the temperature of those rooms nearest the flue, in the upper flats.

This so-called waste of heat is produced in two ways, viz.: (1) by faulty construction of fireplace and grate, and (2) the mode of consumption of fuel. The air-supply of the fire comes from the outside by doors and windows. In ordinary combustion, probably one-third of the coal passes off in smoke, therefore producing no heat, to contaminate the outside atmosphere. This may be called "active" or "quick" combustion, in contradistinction to another mode of fuel consumption called "slow" combustion. In active combustion, the air-supply passes through and from beneath the fire-seat; in slow, it only passes through the fire, and not from below the fire. By reason of the reduced air-supply, in the latter mode, the combustion of fuel is slower, and the chimney-ward movement of air is less rapid; hence, for a smaller amount of fuel, a larger amount of heat is conserved for the uses of the apartment, the fuel lasts a longer time, and the total cost of firing is less. From a long series of practical experiments made by us with different forms of grates, the slow combustion grate has proved the most economical; and the simpler the grate, the better the results. The following form, which has never been patented, has been evolved from these experiments, as it gives the best results. It consists of a plain metal frame, which is "flush" with the wall. The floor is composed of one large thick fire-brick, as are also its sides and back. The outer lining of the fireplace, behind these side and back bricks, is composed of ordinary fire-clay bricks built in cement. The "throat" of the chimney is contracted, and is rounded by cement. In such a grate it is difficult to start the fire. To overcome this a "blower," which consists of a sheet of iron, and which may be pulled down from a sliding socket in the upper part of the grate frame, acts efficiently. When the fire is well started it is pushed back into its rest. Such a fire requires to be replenished but twice daily to thoroughly warm a room of about 6,000 cubic feet capacity. Another advantage of the slow combustion grate is that it does not produce the objectionable draughts between doors and windows and fireplace, present with an ordinary grate. Next to petroleum, coal gives off more heat than any other form of fuel. It gives more than twice that of wood, and when properly burned the carbon unites with the oxygen of air to form carbonic acid gas, thus $C + O_2 = CO_2$.

FIG. 20.—Brown and Green's grate for smoke prevention.

Fig. 20 represents Brown and Green's Smoke-prevention Grate. The coal is put into the fire by a specially constructed shovel below the incandescent coal on the curved ledge shown in the section.

Improper combustion causes smoke, and a smoky atmosphere is the cause of fogs. Domestic fires are the chief cause of the smoky atmosphere of populous cities, and such atmospheres are prejudicial to the public health. The remedy is to be looked for in smoke-consuming grates.

CLOSED STOVES.—These are very commonly used in countries which experience rigorous winters. They economise fuel. They do not, however, warm the room objects so much as the air of the room, neither do they form such efficient outlets for foul air as open fires. They are chiefly made of iron, either solely or with an exterior covering of porcelain slabs or tiles. Cast-iron stoves are by no means impervious to the gases of combustion, as alternate heating and cooling cause expansion and contraction of the metal, and thus the seams or joints are loosened. The chief objection to closed stoves consists in the fact that they "dry" the air of the room; that is, they produce the sensation of dried air upon the occupants. This is by no means imaginary, and is produced in this way. Watery vapour, in gaseous form, is always an atmospheric constituent. As is the temperature of the air at any given time, so is the total possible amount of watery vapour which it can sustain. This is called the maximum amount, or *amount of saturation.* Most usually, however, the air only holds some proportion of this total amount. Most comfort is experienced when the air holds 70 to 80 per cent. of the possible total amount it can hold at that temperature. Suppose, then, when a stove is lighted, that the temperature of the air of the room is 40° Fahr., and it holds 75 per cent. of the total possible watery vapour, or humidity, as it is often termed. When the room temperature has risen to 65 deg. Fahr., the percentage of humidity for that temperature will be less than 75 per cent. of its possible total amount; consequently the air feels dry to the occupants. What happens, then, is this. The air robs everything and everybody in the room of moisture to satisfy its requirements, hence the skin begins to feel dry, the hair becomes crisp, and a general sense of uneasiness ensues. To avoid this it is advisable to place shallow vessels containing water near the stove to supply the needed humidity. But saturation of the air is quickly produced, and when the room air cools down this excess of humidity becomes condensed upon the walls, and is apt to show itself as tiny streamlets. This is most likely to happen when the number of persons in a room is relatively larger than it ought to be.

### Our American Letter.

An impressive little commemorative ceremony took place on Easter Sunday in Woodlands Cemetery, Philadelphia, when the nurses of the Philadelphia Training School assembled at the grave of Alice Fisher. The chaplain conducted a simple service, and the nurses, some sixty of them in all, one by one dropped their offerings on the grave. Miss Fisher's memory is still dear to those who are carrying on the work she began more than ten years ago.

Miss Rebecca West has been appointed chief nurse at the University Hospital, Philadelphia, vice Miss Delana, lately resigned. Miss West was formerly superintendent of the Emergency Hospital, Washington.

The city of Boston can boast of more than one "Nurses' Club." The most important is the "Graduate Nurses' Association," which only, as its name implies, numbers fully trained nurses amongst its members, and aims at providing them, through lectures and clinical observations, with opportunities of keeping in touch with progress in their profession. It has a membership of two hundred nurses. Lectures are given every fortnight, by the courtesy of various physicians, who volunteer their services as lecturers, and a clinic every week in one of the operating theatres of the Massachusetts General Hospital, on the same lines as those given to medical students. The "Nurses' Club" of the Boston City Hospital includes the pupils as well as the graduates of the school. Miss Drown, the superintendent of the training school, is its president. Meetings are held monthly, and there are weekly lectures at the hospital.

The "Ladies' Aid Association," of Lowell, Mass., has undertaken the responsibility of providing funds for the erection of a home and training school for the nurses of the Lowell General Hospital, and is appealing for the necessary money. It is proposed that the new building shall be in the grounds of the hospital.

How to provide adequate care for sick nurses occupied the attention of the Brooklyn Hospital Training School Alumnæ Association at its last meeting. Miss Merritt explained the arrangements which the hospital was making for the future benefit of its nurses in times of illness, in which the members of the alumnæ will participate. This matter is receiving a good deal of attention just now, and a bazaar was lately held in New York by the alumnæ of the Mount Sinai Training School for the endowment of beds for sick nurses. The lady superintendent of the Post Graduate Medical School and Hospital Training School has also been making efforts in this direction, and has appealed for funds to endow a "nurses' ward."

The Arapahoe County Hospital, Denver, has sustained a severe loss by the death of the superintendent of the Nurses' Training School, Miss Josephine Kloth, from typhoid fevers contracted while on active duty. Miss Kloth was one of the first graduates of the Cincinnati Training School for Nurses of 1891, and, during her two years at Denver made herself universally loved and respected.

### Where to Go.

Society for Promoting the Employment of Women, 22, Berners Street, W.—The annual meeting will be held at the office on Wednesday, June 10th, at four o'clock, the president, Lord Stanmore, in the chair. Tea and coffee at 5·15 p.m.

International Life-saving Exhibition, Central Hall, Holborn.—An Exhibition of Manufactures, Appliances, and Inventions for the Saving of Life will be held in aid of Guy's Hospital Re-endowment Fund, from July 9th to 18th.

Stafford House, St. James's.—The concert and dramatic performance in aid of the Free Home for the Dying, announced in this column last week, for June 8th, will be held at Stafford House, by permission of the Duchess of Sutherland, on June 9th, at 3.30 p.m. Mr. Burdett will deliver an address on "Hostels of Peace," at 4.30. The Princess of Wales hopes to be present, and also the Duke and Duchess of York. Tickets one guinea each (two for 30s.) or 10s. 6d. (three for 25s.), may be obtained from Miss Erskine, Speaker's Court, Palace of Westminster.

Exhibition at Messrs. Dowdeswell's Galleries, 162, Bond Street.—A small but interesting collection of pictures is now on view at Messrs. Dowdeswell's galleries. These are some representative works of deceased masters of English and foreign schools, which includes a Reynolds, Romney, Janssens, Morland, and last, but not least, a Ruijsdael; it speaks, therefore, for itself that the present exhibition is well worth a visit, and many of the canvas are representative of the master hands which painted them. Portraiture is the most conspicuous element, but landscape and subject pictures are not wanting. Among the portraits, John Opie's "Miss Cooper" finds a prominent place, perhaps the most prominent in the gallery. It is a three-quarter length canvas of a fine young lady, sister of Lady Waterpark. Being painted early in the nineteenth century, the dress, and its treatment, are suggestive of the epoch following closely on Sir Joshua's. The vivid life-like colouring of the face is masterly, as is also the delicate treatment of the texture of the veil which drapes the figure. It is a remarkably well-preserved picture, and shows John Opie at his best. No. 17, "Age and Infancy," by the same brush, does not strike one in the same insistent manner which the brilliant "Miss Cooper" does; this was exhibited at the Royal Academy in 1783. George Romney's "Portrait of a Gentleman," No. 1 in the catalogue, is not well known to the public, and is of interest on that account, but Romney's style lent itself more readily to female portraiture. The porcelain-like delicacy of C. Von Vos' painting of "The Sisters" is noticeable; equally finely painted is a small subject picture of Dirk Hals. This is a lively and bright study of "A Luncheon Party"; perhaps a little too small in its dimensions. "Meditation," by Sir Joshua Reynolds, an old friend to many of us, but one which ever improves on acquaintance, is here in all its loveliness, mellowed and beautified by time. The Ruijsdael is the great attraction at Messrs. Dowdeswell's present collection; it is an upright landscape study in Holland. This fine specimen was in Baron Verstock's collection; it was painted in 1681, and is in wonderful preservation. Dutch art is shown at a high level in this example, as, indeed, it is in the other canvas in this exhibition.

### On Certain Aspects of the Nursing Question as Seen in England and Germany.

By a Certificated Midwife.

### IX.—A PATIENT'S EXPERIENCES IN GERMANY.

To be ill in a strange land, to be taken to a strange hospital, and to lie there for weeks, seeing no one but doctor and nurse, sounds a dismal tale. Looking back on it after nearly twenty years, I consider the experience gained then to have been of the highest value to me. My illness was enteric fever, and as I made a most excellent recovery under the care of one of the kindest and cleverest of doctors, a little account of the way I was nursed may be of interest to my readers.

The complaint which had attacked me is a sadly common one. Hardly a family but knows something of its ravages, and of the dreary train of weakness and morbid conditions which generally follow on convalescence. I had had much to do in England with the nursing of typhoid cases. I had lost a sister myself from this disease, and I knew a good deal of the English methods of treatment, and so when I arrived at Ludwig's Spital and was taken upstairs I speculated in a dull indifferent kind of way as to whether I should ever come down again.

This hospital, of which my good doctor was the *Vorstand*, was not a large one. I think it contained from 50 to 60 patients. It was comparatively new, and had been built by the bequest of a deceased physician of the town. It stood in a quiet street just off the line of general traffic, and was surrounded by a small garden. From the windows one could look out on the lovely vine-clad hills which frame the picturesque city of Stuttgart. It was built in three storeys. The lower one was occupied by the *personnel* of the establishment, and contained also the consulting-room and the private apartments of the house surgeon. The second floor was allotted to male patients, and the third to female ones. Below all, though not really underground, were the kitchens and engine-room, and all the heat of the house emanated from this latter, whether it was wanted for cooking or warming.

Three classes of patients were admitted to the benefits of this hospital, and all had to pay something, whether personally or through the subscription of the *Verein* to which they belonged. The first class had each a room to themselves, and, fortunately, this luxury was mine; but as there was not one vacant on the third floor I was accommodated with one on the second in the men's division, but of this I knew nothing till I was able to walk about again. The entire nursing was undertaken by the deaconesses, who had a large institution of their own not far off. I think there were five sisters in charge when I was there, but Sister Röslie and Sister Trinette were the only two I came in contact with. They were entirely under the orders of Dr. Teuffel while there, but were overseen by the mother of their own institution and changed at her discretion. They were trained at their own infirmary, and I believe the system was that a certain sum was paid yearly to the *Diaconnissen Institut*, and that it undertook to supply as many nurses as were wanted. Anyhow, the nursing seemed to run on greased wheels. Sister Röslie it was who chiefly attended me, with Sister Trinette to superintend and take the night watches at times. Good little Sister Röslie! How well I can recall her homely, rosy face, her dumpy little figure clad in the uniform of her order; the black stuff dress and round tippet of the same; the large blue chequed apron with its pattern of carefully ironed creases; her plain linen cap tied with tape strings.

I do not think, by the way, that any sentimental young ladies in Germany would be attracted into the nursing profession by the becomingness of its costume, as I have heard it rumoured is now and again the case amongst us! As for my little nurse, she had no sentiment about her, nor did she make any pretensions to an angelic status, but for all that she was a ministering spirit. I owed her much, for she was conscientious and faithful to a degree, untiring in her attentions, and always smiling. I gave her my photograph when I left because I was absolutely forbidden to give, or she to receive, anything else. I wonder if she keeps it still as I treasure hers? I look at it sometimes and think of the stories she used to tell me of her work amongst the poor when I was convalescing and able to sit up. "Sister," said I, one day, "which do you like best—nursing in a hospital, or private cases whether poor or rich?" "Oh, Fräulein, there is no comparison to be made. Here we have everything we want both for ourselves and our patients. There is no lack of food or firing, and the good doctor is always at hand with his medicines; it is all easy and pleasant, and then our pride rises and we think how clever we all are. And so it is in the houses of the rich. They think money can do everything, and are cross if it does not bribe death to pass them by. But when one goes to the poor, and finds the mother ill in bed, and the father away earning barely enough to find the coarsest food, and there is no fire unless you go into the wood to gather sticks, and there is no water unless you fetch it, and the children are crying with cold and hunger, and you have no medicines at hand, and no doctor at your side to direct you, then it is one feels the blessedness of being a nurse. There is no one to help but *der liebe Gott*, and so one finds He is close at hand. He softens the hearts of the neighbours when you go to beg for a drop of soup or a faggot of wood. He sends you a few pennies out of one purse, and some clean linen out of another cupboard; and whether the sick one dies or lives there is no room for pride, and no murmuring at His will."

Besides the five sisters, there were servants downstairs who did the cooking, but I never saw them. Cleaning there was none to be done in the wards, and of all the attributes of a German hospital this was, I think, what struck me with the greatest sense of contrast as I lay there a patient myself. I remembered the scrubbing and the dusting of our London wards; the damp floors, the heavy air, reeking with the smell of carbolic; the black smoking fires, the soot and fog coming in at the windows when they were open, and the sad, suffocating feeling if they were shut. And when my fever permitted me to think at all, I thought what a delightful change was the spotless polished floor, and the clean, warm air which found its way into the room from tubes connected with the engine-room, and purified by passing through cotton wool, as I afterwards learned. But there is little dirt or dust in winter when the snow lies on the ground, and in a city where the fuel used is wood, and the stoves are all close ones; and sometimes I persuaded the sister to let me have one of the double windows open, which she thought would certainly kill me, till the Herr Doctor, with his indulgent smile at an Englishwoman's fancy for the open air, bade her remember I was an *Ausländerinn*, and accustomed to cold.

(*To be continued.*)

### Bolingbroke Hospital, Wandsworth Common.

The annual garden fête and sale of work in aid of this hospital will be held on Saturday afternoon, June 6th, in the grounds of the hospital. Admission, half-past two to six p.m., 1s.; six to ten p.m., 6d.

### The Visiting Nurse.

(By a Correspondent.)

For some years past two or three hospital-trained nurses, leaving the beaten track of ordinary private work, have been quietly pursuing the calling of daily or visiting nurses. So much has been brought to light by the correspondence on the subject that has lately appeared in these columns. The advantage to the public of this special class of nurse has not as yet been fully acknowledged, but the development of the movement is simply a matter of time. At present there are but few workers in this comparatively new field. Those who have most need of the aid of the daily nurse have been for the most part unaware of her existence, and the general practitioner to whom she must look for approval and work has not had time to consider the matter in all its aspects.

The duties of the visiting nurse resemble those of the district nurses, with the exception that the patients of the former belong to a somewhat different class. She is much less confined than at ordinary private nursing, getting rather too much than too little of outdoor exercise. If she is active, both as regards her work and in getting from place to place, she is able to have several cases on her list at the same time. If she is fortunate enough to find all her patients for the time being in the same locality, she can increase the number, but as a rule she finds it difficult to give the needful daily attention to more than three sick persons. In a great measure this depends on the attendance required by each patient, the duration of the visit varying from half-an-hour to an hour, and even longer. Sometimes there is much forethought wanted to make the cases fit in with each other—the demand for the hours between eight and eleven being greater than for all the rest of the day.

The work done by the daily nurse is usually that which cannot be undertaken by the friends of the patient. Acting under the directions of the medical attendant she gives enemata and douches, applies poultices, blisters, fomentations, and renews dressings in cases of less important surgical operations. In many instances where other nursing is not required, it is arranged for her to come in to wash and make the patient comfortable for the day, and she returns in the evening to perform the same services for the night. It is usual for her to carry a bag containing those articles that may be wanted, such as a good syringe, catheter, &c.; it must contain also some lint, bandages, glycerine, and some kind of disinfectant. The ordinary midwife bag, with some addition to its contents, will give an idea of what is required.

After the day's work is done it is usual to leave a written report at the door of the medical attendant under whom the nurse is working. Should she have anything particular to say to him she waits for a personal interview. In some cases he will let her know the probable time of his visits, so that she may arrange, if possible, to meet him at the patient's house to give him any help he may require, or to receive his orders. The work of the daily nurse is greatly facilitated by working under one medical man only.

To those people of small incomes who naturally shrink from gratuitous aid and are yet unable to afford a resident nurse, the comfort of being able to obtain trained assistance that comes within their means is inestimable. There are many cases in which the friends of the patient are competent to undertake the general nursing of the invalid, but are unable to manage those things that require the practised hand of the skilled nurse. To these may be added the dwellers in flats, who in cases of illness are much limited as regards air and space; the extra room and extra attendance necessitated by a nurse living in the house is almost an impossibility. Many invalids, bearing with what patience they can the kind, but inexperienced handling of friends, would be glad to know that they could obtain the help they need without the inconvenience caused by the constant presence of the nurse.

To those nurses who prefer to live in their own homes, even at the risk of decreased earnings, this branch of work offers many attractions. It gives an opportunity to the highly-strung but often excellent nurse who sinks under the nervous exhaustion entailed by night duty. The mental strain is also relieved by the fact that the more serious cases, both medical and surgical, will always require the services of a resident nurse. A few hours' rest can be often obtained in the middle of the day owing to the greater part of the work being crowded into the morning and evening.

The qualities required in addition to thorough training are the quickness of eye that will notice at a glance what has to be done, and the deftness and method that will do it well in the limited space of time at disposal.

The earnings of the daily nurse vary greatly. The ebb and flow of cases that affect all branches of nursing alike diminish her opportunities of regular work; she has either too much or not enough to do. Roughly, her income may be put down as averaging £80 to £90 per annum. Out of this she has, of course, to pay rent and food. The terms charged per visit vary also. In most cases the payment is half-a-crown per time, a reduction being sometimes made if the nurse's services are required twice daily. The usual small expenses of omnibus or train are discharged by the patient. In cases of illness that are likely to last some time, the nurse is often paid by the week instead of by the visit.

At present the demand for daily nursing is not greater than the supply. It requires time and publicity to obtain the recognition of its undoubted value. Those nurses who wish to make an opening for themselves must be prepared with a little capital for the time of waiting and much perseverance. They must make up their minds also to have plenty of patience—a good daily round of nursing cannot be obtained all at once. It is fatal to get tired of waiting and to take a resident case to fill up time—few medical men will send twice to a daily nurse who cannot be depended on to be at home at certain hours in the twenty-four. The nurse who wishes to make a move in this direction should consult some general practitioner to whom she is well known, and ask for his assistance and encouragement. As a rule two visiting nurses living and working together have a much better chance of getting on than one who depends entirely on herself.

### Exhibition of Nursing Appliances.

An exhibition of nursing appliances, organised by Mrs. Bedford Fenwick, editor of the *Nursing Record*, was opened on Monday, June 1st, at St. Martin's Town Hall, Charing Cross. The large hall is devoted to purely "nursing" exhibits, which include contributions of padded splints, all varieties of special dressings and bandages, poultice jackets, ward baskets, and models of beds, operation tables, and other ward and sick-room paraphernalia. The nurses of the Homœopathic Hospital are much to the fore with model cots and dolls, beautifully got up, and the Mildmay Hospital nurses also shine in the matter of dainty contributions. A central place in the hall is occupied by the model of a nurse in specially made costume, mounted on a bicycle. In the smaller hall, specimens of foods and tonic wines, nursing uniforms, &c., are exhibited.

On Wednesday, Thursday, and Friday papers on various nursing matters were read by Miss Mollett, Miss Isla Stewart, Miss Frances Hughes, Miss Margaret Breay, and others, in the Lecture Room on the ground floor. A conversazione took place on June 3rd, and another will be held on Saturday, the 6th.

### Dress and Uniforms.

By a Matron and Superintendent of Nurses.

### THE LONDON TEMPERANCE HOSPITAL.

This bright pretty little hospital stands out like an oasis in the desert of the dreary Hampstead Road. The home-like comfortable appearance of the wards is in curious contrast to the uninviting surroundings of the neighbourhood, and once within the portals one forgets the dingy streets that have just been left outside. Pleasant-faced nurses in neat uniforms are seen gliding to and fro between the beds, and as we stop to admire them we notice that the sister of the ward is attired in green alpaca, a material non-absorbent of dust, and consequently most suitable for the purpose. The dress is made full and plain, just clearing the ground, and is gathered at the waist into a band, to which the bodice is attached. A white linen apron of ample width is worn over this, which is completed with a square bib and straps which cross behind and button at the waist. The neat little cap is of the shape known as the "Sister Dora," and ties under the chin in a compact bow. White lawn is the material of which it is made, and the effect is very light and dainty. Linen cuffs and collars make a becoming finish to the neck and wrists. The staff nurse wears a costume of light blue cambric made quite plain, the bodice buttoning in front. An apron of white linen with bib and straps, and provided with pockets, comes next, and the cap, which is of the becoming coronet shape, is made of cambric and edged with two rows of Cash's Coventry frilling. The edging is put on full and gophered, the gophers being kept in position by a thread run through them on the reverse side.

The illustration portrays a probationer, whose costume varies from the staff nurse in the matter of the dress only. To make the necessary distinction the former wears blue and white striped regatta. It is made with a full plain skirt, and bodice, fastening in front. This material is always clean and fresh looking, and washes so well that we trust its large and deserved popularity will be maintained for a long time to come.

### Everybody's Opinion.

[Correspondence on all subjects is invited, but we cannot in any way be responsible for the opinions expressed by our correspondents. No communications can be entertained if the name and address of the correspondent is not given, or unless one side of the paper only be written on.]

### THE TRUTH OF THE MATTER.

**Three Years' Training for Nurses.**

"A London Matron" writes: I cannot resist writing to tell you how pleased I was to see the excellent paragraph in this week's Hospital Supplement headed "The Truth of the Matter." It deals with the subject fairly and reasonably all round, and I quite agree with you that it is a truth that needs to be plainly stated, and made as widely known as possible. Among the many fallacies that are constantly being impressed upon the general public in regard to nurses the "time" theory concerning the training of nurses seems to me one of the most mischievous. The conditions for acquiring a sound knowledge of nursing, the varied and very different objects with which training is sought, the extreme contrast between the individual capacity of those who enter hospitals for the purpose of gaining the necessary technical qualifications to fit them for all sorts of positions in the hospital or nursing world; all these vital considerations are apparently lost sight of, or, at any rate, are allowed to sink into insignificance beside the one parrot-like cry of "three years' training." As usual in such cases a penalty has to be paid all round for the setting up of a false standard by which to measure the qualifications demanded. I agree with your article that it is perfectly fair that a three years' engagement should be customary. The probationer owes some service to her hospital for the training received, and there is obvious risk that the quality of that training might gradually deteriorate unless a good proportion of trained workers were retained on the hospital staff. But the point is that the extra time fairly comes under the heading of experience, and that after two years' hospital training all further work should rightly be regarded as experience, and, as far as may be, adapted, of course, to the best sort of preparation for the particular work ultimately in view. It was so refreshing to have facts, with which we are all so familiar, thus frankly stated that I feel bound to tell you how glad I am to see "the truth of the matter" definitely set forth. Those who read your paper without prejudice can scarcely fail to perceive its justice, but doubtless hundreds of nurses, not to speak of the public, have yet to suffer before the fallacy of the "three years' training" becomes generally understood.

### ENGLISH CONVALESCENT HOMES.

"A Lady Superintendent" writes: In your issue for May 23rd, "Marah" suggests that anyone requiring admission to a convalescent home for a patient should write in the early stages of the illness to the Home authorities stating nature of illness and asking for the probable date of a vacancy—that the letters could then be dated for admission, and if the patient can wait for the vacancy, nothing but a letter before their arrival to the matron would be necessary. I should like to say that my experience as superintendent of a convalescent home is, that perhaps one hundred people a year will write asking when such and such a patient can be taken in, and when given a date either take no further notice, or, as often happens, ask for vacancies to be kept, and do not send the patient or even an explanation of non-arrival. This leaves empty beds, when a hundred or more applicants are waiting to come in, and gives a great deal of unnecessary trouble to the authorities. In the Home of which I am superintendent during three months of this year no less than forty expected patients failed to come.

### Notes and Queries.

**Queries.**

(58) *Electrolysis.*—Can you tell me if electricity is the best and most permanent cure for superfluous hair, and if so, recommend a good operator?—*K.*

(59) *Post-mortem Assistant.*—Will you let me know, through the columns of The Hospital, how an assistantcy in the post-mortem department of a hospital can be obtained?—*Constant Reader.*

(60) *Holidays in the Channel Isles.*—Can you or any readers of The Hospital give me information regarding the best route from Glasgow to Alderney or Sark, and the probable cost of lodgings and food there for two nurses in August?—*Sister.*

(61) *Paralysis.*—Where could a person suffering from paralysis of the right arm be treated as an out-patient?—*West Kensington.*

(62) *Cubic Space.*—How much cubic space should be allowed for two patients suffering from measles, and a nurse in attendance?—*Sister May.*

(63) *Monthly Nursing.*—Information wanted as to the duties of a monthly nurse.—*Nurse R.*

(64) *Advice Wanted.*—Can you tell me how it would be possible for a lady, a trained and certificated nurse, to add to a small income where her husband could be with her?—*Matron.*

(65) *Guild of St Veronica.*—Where should I apply for information concerning the Guild of St Veronica?—*Veronica.*

(66) *Homes for Paralytic Cases.*—Can you tell me at what institution a case of paralysis could be received? Terms must be very moderate.—*Mrs. M. N.*

(67) *Co-operation.*—Is there any Nurses' Co-operation in Dublin?—*Erin.*

(68) *Burdett's "Hospitals and Charities."*—Please tell me how to get particulars of a hospital inserted in Burdett's "Hospitals and Charities."—*Enquirer.*

**Answers.**

*Holland Institute* (*K. M. J.*).—No notice can be taken of anonymous communications.

(58) *Electrolysis* (*K.*).—It is a treatment largely practised now, and your own medical man would be the proper person to whom to apply for advice.

(59) *Post-mortem Assistant* (*Constant Reader*).—Such vacancies are usually filled privately. Your only course would be to watch advertisements and to advertise yourself.

(60) *Holidays in the Channel Isles* (*Sister*).—Write to Messrs. Cook and Son, Ludgate Circus, E.C. The route they recommend is by Midland Railway to London, and thence by Great Western *viâ* Weymouth, or London and South-Western *viâ* Southampton. Fares from Glasgow to London and back, available to December 31st, £5 10s. 3d. first class, £2 12s. third class. Return ticket, London to Guernsey, £2 8s. first class, £1 18s. second class, available for two months. For all further particulars read "A Holiday in Sark" in The Hospital Nursing Supplement for May 12th, 1894.

(61) *Paralysis* (*West Kensington*).—You will find full lists of hospitals and institutions in Burdett's "Hospitals and Charities." (Scientific Press, 428, Strand, W.C.)

(62) *Cubic Space* (*Sister May*).—In most hospitals for infectious disease 1,500 cubic feet are allowed per patient. At this computation 4,500 feet would, therefore, be allowed for two patients and a nurse.

(63) *Monthly Nursing* (*Nurse R.*).—Read the chapters on Monthly Nursing and Midwifery in Dr. Percy Lewis's "Nursing: Its Theory and Practice," Scientific Press, 428, Strand, W.C.

(64) *Advice Wanted* (*Matron*).—You might undertake the care of an invalid; we can only suggest that you should watch the advertisements, and let your friends know that you would be glad to receive such a case. Would you care to provide a home for an elderly lady; and, if so, on what terms?

(65) *Guild of St. Veronica* (*Veronica*).—Write to Miss F. Robertson-Macdonald, 9, Great Bedford Street, Bath, the secretary of the Guild.

(66) *Homes for Paralytic Cases.*—See reply above under "Paralysis."

(67) *Co-operation* (*Erin*).—We do not know of any.

(68) *Burdett's "Hospitals and Charities"* (*Enquirer*).—Send the particulars you wish inserted addressed to The Editor, Burdett's "Hospitals and Charities," 428, Strand, W.C.

We regret that want of space will prevent many queries from being answered this week. They will be taken in rotation in due course, and attended to as promptly as possible.—Ed. T. H.

## Figures and Tables

**Fig. 20. f1:**
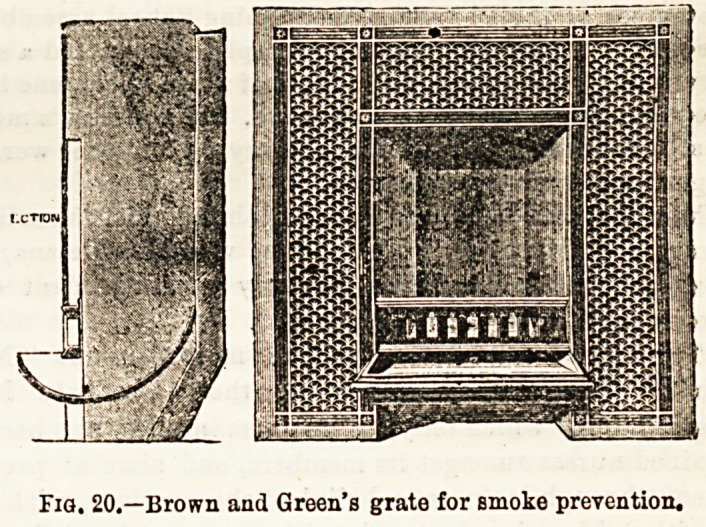


**Figure f2:**